# Haloperidol treatment induces tissue- and sex-specific changes in DNA methylation: a control study using rats

**DOI:** 10.1186/1744-9081-2-37

**Published:** 2006-11-29

**Authors:** Morihiro Shimabukuro, Yoshihiro Jinno, Chiaki Fuke, Yuji Okazaki

**Affiliations:** 1 Department of Molecular Biology, Ryukyu University School of Medicine, Okinawa, Japan; 2 Department of Legal Medicine, School of Medicine, University of the Ryukyus, Okinawa, Japan; 3 Department of Psychiatry, Mie University School of Medicine, Mie, Japan

## Abstract

**Background:**

We previously found that there is a subtle difference in the global methylation state of blood leukocyte DNA between male subjects with and without schizophrenia. The aim of the current study was to determine whether this difference was a primary effect of the disease state, or a secondary effect of antipsychotics administered to these patients.

**Methods:**

We examined the methyl cytosine (mC) content of DNA from the leukocytes, brain, and liver of rats using high performance liquid chromatography. A total of 40 male and female rats received for 21 days daily injection of haloperidol or vehicle solution alone.

**Results:**

In control rats injected with buffer only, there was a sex-dependent difference in mC content in leukocyte DNA (male > female; *P *= 0.028, n = 10), similar to our previous observations in human peripheral leukocytes. No difference in mC content between the sexes was observed in the brain or liver in buffer-treated animals. Haloperidol treatment slightly decreased the mC content of leukocytes in male rats, but unexpectedly, increased the mC content of leukocytes in females. We observed a trend toward a higher level of mC in the liver in both sexes following haloperidol treatment, compared to buffer-treated animals. In contrast, haloperidol treatment resulted in a decrease in mC content in the brain in females, and this difference was statistically significant (*P *= 0.026).

**Conclusion:**

These results indicate that haloperidol can affect DNA methylation states in the brain, as well as in certain other tissues, and raise the possibility that antipsychotic drugs play a role in the observed disparity in mC content in male subjects with and without schizophrenia.

## Background

Epidemiological studies have established that genetic factors play a major role in the development of schizophrenia. However, the discordance rate for schizophrenia between monozygotic twins is approximately 50%, suggesting that epigenetic and/or environmental factors are also involved in the development of the disease. Despite extensive research, the molecular etiology of schizophrenia remains enigmatic [1,2]. The use of microarrays or DNA chips for genome-wide analysis of gene expression is showing that the underlying variation in gene expression among individuals may contribute to the development of complex traits and characteristics [3,4]. Epigenetic modifications, such as DNA methylation, play an important role in the regulation of gene expression, primarily through their role in regulating chromatin structure and function [5]. Defects in epigenetic factors are linked to several diseases [6,7]. For example, Rett syndrome, a neurodevelopmental disorder, is caused by mutations in the gene encoding methyl-CpG-binding protein-2 (*MECP2*) [8], and alpha-thalassemia/mental retardation X-linked (ATRX) syndrome is caused by mutations in *ATRX*, which encodes a member of the SWI/SNF family of chromatin remodeling proteins. Patients with ATRX syndrome exhibit severe mental retardation as well as alpha-thalassemia [9].

Unlike Rett and ATRX syndromes, symptoms of schizophrenia appear later in life, suggesting that environmental factors contribute to the development of the disease. The methylation state of the genome undergoes highly dynamic changes, extensive demethylation and reconstruction during early embryogenesis, yet once established, is very stable [10]. Nevertheless, some epigenetic signals, including DNA methylation, can be transmitted from one generation to the next, and are influenced by environmental or intrinsic biological factors [11-14]. Thus, DNA methylation and/or other epigenetic modifications of the genome may help explain the ambiguity of inherited schizophrenia and the role, if any, of environmental factors in the etiology of the disease.

In developing a model of schizophrenia [15], we examined global methylation of peripheral leukocyte DNA from more than 200 patients with schizophrenia, and compared it to global methylation in healthy subjects. The results revealed lower mC content in male patients than in male controls, although the difference did not reach a statistically significance [16]. In the present study, we were interested in determining whether this difference was a secondary effect of anti-psychotic medications.

## Methods

### Animals and chemical

Eight-week-old male and female Sprague-Dawley rats (330 – 360 g and 190 – 230 g, respectively) were purchased from Kyudo Co., Ltd. (Kumamoto, Japan), housed in standard cages in a controlled environment at a constant room temperature (24 ± 2°C) and humidity (50–70%), and maintained on a 12 h light-dark cycle. Food and water were provided *ad libitum *through out the experiment. Haloperidol was kindly provided by Dainippon Sumitomo Pharma Co., Ltd. (Osaka, Japan). The bulk powdered chemical was dissolved in 0.1 M glacial acetic acid buffered to pH6.0 with NaOH at concentration of 0.25 mg/ml [17]. All animal experiments were reviewed and approved by the Ryukyu University Animal Welfare Committee.

### Experimental procedure

Ten male and 10 female rats were administered 0.5 mg/kg haloperidol intraperitoneally on a daily basis [18]. Control rats received the same volume of vehicle solution alone under parallel conditions. After 21 days of daily injection, the rats were sacrificed by injection of sodium pentobarbital (120 mg/kg) intraperitoneally. Trunk blood was collected using a heparinized syringe, and plasma was prepared by centrifugation at 2,000 rpm for 10 min, then stored at -30°C until use. Layered cells, which include whole white blood cells, following centrifugation were isolated and set aside for DNA extraction. Whole brains, excluding the cerebellum and olfactory bulb, liver, and kidney were rapidly excised, rinsed twice in saline, then snap-frozen in liquid nitrogen.

### HPLC analysis

mC content in blood leukocyte DNA was evaluated using high performance liquid chromatography (HPLC). All reagents and conditions for HPLC analysis have been described previously [19]. Global methylation levels are expressed as the ratio of mC to the sum of 2'-deoxycytidine and mC (%).

### Statistical analysis

Statistical analyses were performed using Stat View software (SAS Institute Inc., Cary, NC, USA). Differences in mean mC content between the two groups were evaluated with the Mann-Whitney U test. Results were considered statistically significant when the *P *value was less than 0.05.

## Results

### Physiological state of haloperidol-treated rats

All rats treated with haloperidol grew as well as control rats and did not exhibit noticeable abnormal behavior during the course of the experiment. Body weights and blood concentrations of haloperidol and prolactin at the time the animals were sacrificed are summarized in Table 1.

**Table 1:** Body weight and plasma concentrations of prolactin and haloperidol for control and haloperidol-treated rats

Sex	Treatment^a^	Body weight (g) at		Plasma concentrations of	
		beginning	end	prolactin (ng/ml)	haloperidol (ng/ml)
Male	haloperidol	351.5 ± 6.9	476.0 ± 8.4	64.7 ± 43.7	4.3 ± 0.9^b^
	placebo	342.5 ± 6.3	473.0 ± 9.5	37.3 ± 10.0^b^	1.5, 2.0, <1.3^c^
					
Female	haloperidol	211.0 ± 15.2	298.0 ± 16.9	441.1 ± 243.3	ND
	placebo	211.0 ± 12.9	300.0 ± 15.6	150.5 ± 112.7^b^	ND

^a^Each group consisted of 10 rats.

^b^n = 5

^c^Haloperidol was below the detection limit in three of the five rats examined.

ND: not determined due to a high background activity of unknown origin.

Plasma concentrations of prolactin and haloperidol were determined by the [^125^I]-labelled radioimmunoassay and colloidal gold immunoassay, respectively.

### Sex- and tissue- specific global methylation levels in control rats

In a previous study, we found a sex-dependent difference in the mC content of human leukocyte DNA, with the level in males being greater than that in females [16,19]. In the current study, we first compared the mC content of leukocyte DNA in male and female control rats. There was a statistically significant difference in the mC content of leukocyte DNA (males greater than females), which was consistent with our previous results in humans. However, there was no difference in the mC content between males and females in either the brain or liver DNA (Table 2). The differences in global methylation levels among different tissues were quite remarkable (leukocytes > brains > livers).

**Table 2:** Tissue- and sex-specificity of methylcytosine (mC) content (%) in control rats

	mC content in leukocyte, brain, and liver DNA (mean ± SD)					
	
Sex (n)	Leukocytes	*P*	Brain	*P*	Liver	*P*
Male (10)	3.843 ± 0.042	0.028	3.650 ± 0.023	0.791	3.412 ± 0.033	0.496
Female (10)	3.771 ± 0.048		3.652 ± 0.031		3.426 ± 0.027	

n: numbers of rats analyzed.

Differences in mean mC content between male and female rats were evaluated with the Mann-Whitney U test.

### Effect of haloperidol on global methylation levels in leukocytes, brain, and liver

We next compared the mC content in haloperidol-injected rats (drug group) to buffer treated control animals (control group) by sex and tissue type. In males, the mean level of mC content of leukocyte DNA was slightly lower in the drug group than in the control group, but the difference was not statistically significant. In contrast, in female leukocytes, we observed a trend toward higher mC levels in the drug group compared to the control group, although again, the difference did not reach a statistically significant level (*P *= 0.064). Thus, the sex-dependent difference in mC content of leukocyte DNA between male and female rats appeared to be ameliorated by haloperidol treatment. Global methylation in the brains of haloperidol-treated rats was lower than in the brains of female control animals, and this difference was statistically significant (*P *= 0.026). There was a higher level of methylation of liver DNA in the drug group compared to the control group, for both males and females, and in males, this difference was statistically significant (*P *= 0.013). These results are summarized in Table 3. No statistically significant correlation between prolactin concentration and mC content in any tissue was detected (Spearman's ρ = -0.104, *p *= 0.570 in leukocytes; ρ = -0.220, *p *= 0.205 in the liver; ρ = -0.193, *p *= 0.279 in the brain).

**Table 3:** Effects of haloperidol on the mC content of leukocyte, brain, and liver DNA

		mC content (mean ± SD)					
		
Sex	Treatment^a^	Leukocytes	*P*	Brain	*P*	Liver	*P*
Male	Haloperidol	3.817 ± 0.036	0.212	3.632 ± 0.047	0.273	3.445 ± 0.027	0.013
	Placebo^b^	3.843 ± 0.042		3.650 ± 0.023		3.412 ± 0.033	
							
Female	Haloperidol	3.812 ± 0.050	0.064	3.615 ± 0.031	0.026	3.449 ± 0.055	0.089
	Placebo^b^	3.771 ± 0.048		3.652 ± 0.031		3.426 ± 0.027	

^a^Each treatment-group consisted of 10 rats.

^b^injected with solvent, NaOH-buffered acetic acid (pH6.0), alone.

Differences in mean mC content between haloperidol- and placebo-treated groups were evaluated with the Mann-Whitney U test.

## Discussion

The aim of the current study was to determine whether global hypomethylation of peripheral leukocyte DNA in male patients with schizophrenia was a secondary effect of their medication. As a model system for studying this question, we chose haloperidol-injected rats, since haloperidol, until recently, was a commonly used drug for the treatment of schizophrenia. In leukocyte DNA from control rats that were injected with buffer alone, there was a sex-dependent difference in mC content (females lower than males), similar to what was previously observed in humans [16,19] The apparent paradox of a lower methylation state in females may be partly attributed to global hypomethylation of the inactive X chromosome [20]. The global methylation level in rat liver and brain was also consistent with what has been observed in humans: liver < brain [19]. Thus, the data obtained from rats and humans are comparable. In male rats injected with haloperidol, the amount of mC in leukocyte DNA was less than that seen in control male rats, but the difference was not statistically significant. Although further study is needed in order to fully understand the difference in mC content of peripheral leukocyte DNA between male patients with schizophrenia and healthy male subjects, the results of the current study present several interesting and novel findings, and implicate a role for haloperidol, and perhaps other antipsychotic medications, in the alteration of DNA methylation in schizophrenic male patients.

Haloperidol-treatment resulted in a decrease in the amount of mC in leukocyte DNA in male rats, although the difference did not reach a statistical significance. In contrast, we found a trend toward higher levels of mC in leukocyte DNA in female rats treated with haloperidol (*P *= 0.064). Although the reasons why haloperidol treatment would have opposite effects on global methylation in male and female rats are not clear from this study, it is possible that these differences are the result of disruptions in the balance of hormones in these animals. This is supported by observations that there are sex-dependent differences in the immune response to certain hormones, and in the frequency of occurrence of some autoimmune diseases [21-23]. It is also possible that the observed effect might be mediated by the alteration in the subset profile of white blood cells. The partial discrepancy between the results obtained from patients with schizophrenia and from rats injected with haloperidol will require additional study, in particular into the differences between humans and rodents in the regulation of epigenetic factors, or other systems that may effect the course of disease [24,25].

In the liver, haloperidol increased global DNA methylation in both males and females, and the difference was statistically significant in males (*P *= 0.013). In the brain, haloperidol treatment resulted in a decrease in females, and the decrease was statistically significant (*P *= 0.026). The differential effect of haloperidol on DNA methylation in various tissues suggests that the regulation of global DNA methylation by this drug occurs through multiple, indirect pathways. In these pathways, sex-specific hormones may play a role in modifying the methylation state of DNA, since haloperidol also disturbs the normal regulation of sex-hormone secretion.

Several studies have established that there are gender differences among patients with psychiatric disorders, suggesting that sex-specific hormones play a role in the pathogenesis of these disorders, including schizophrenia [26-29]. It remains a topic of considerable interest whether and how these hormones are involved in disease progression. Clinical phenotypes are most frequently the manifestation of multiple alterations in genetic and environmental factors. In the current study, using a limited number of rats, we were unable to demonstrate a causative effect of haloperidol on the observed hypomethylation of leukocyte DNA in male patients with schizophrenia [16]. However, we did uncover several sex- and tissue-specific effects of haloperidol on DNA methylation. Of note, valproate, a drug that is commonly used to treat bipolar disorder, has been shown to have a demethylating effect [30]. Clarification of the cellular pathways that mediate haloperidol's effect on DNA methylation state will help elucidate the molecular etiologies of schizophrenia.

## Conclusion

In the current study, using a limited number of rats, we were unable to demonstrate a causative effect of haloperidol on the observed hypomethylation of leukocyte DNA in male patients with schizophrenia. However, we did uncover several sex- and tissue-specific effects of haloperidol on DNA methylation, which helps the researchers interpret the data in epigenetic studies in schizophrenia, and also would shed light on the mechanism of action of antipsychotics.
